# Green design in living and bedroom spaces: exploring environmental restorativeness and affective qualities of spaces

**DOI:** 10.3389/fpsyg.2025.1631417

**Published:** 2025-10-31

**Authors:** Silvia Bellini, Laura Miola, Alessandro Sperduti, Alessia Caccaro, Enrico Pinton, Monica Graffeo, Francesca Pazzaglia

**Affiliations:** ^1^Department of General Psychology, University of Padua, Padua, Veneto, Italy; ^2^Department of Mathematics “Tullio Levi-Civita”, University of Padua, Padua, Veneto, Italy; ^3^Human Inspired Technologies Research Center – HIT, University of Padua, Padua, Veneto, Italy; ^4^Caccaro SRL, Villa del Conte, Italy; ^5^Studio Monica Graffeo, Cordenons, Italy

**Keywords:** biophilic design, restorative design, residential environments, indoor plants, nature

## Abstract

**Introduction:**

Previous research has suggested that the introduction of greenery into built environments has positive effects on individuals' physical and psychological well-being. However, these studies have primarily focused on public spaces, overlooking domestic environments. Additionally, different types of greenery and the influence of individual differences in environmental evaluations have not been adequately considered, all of which are the main objectives of this study.

**Method:**

A total of 331 adults (18–67 years old) evaluated various images of domestic interiors (bedrooms and living rooms) designed with different furnishing conditions (no greenery, with potted greenery, and integrated greenery), rating perceived restorativeness and the affective qualities of the spaces.

**Results:**

Results showed that rooms with greenery were perceived as more restorative and associated with more positive affective qualities. The direct integration of plants into the furniture made the spaces more fascinating and less monotonous, but also more chaotic and less coherent compared to the use of potted greenery. Furthermore, individuals with higher openness to experience and a stronger connection to nature tended to evaluate environments as generally less chaotic.

**Discussion:**

Overall, the findings demonstrate that a biophilic design approach enhances perceived restorativeness and the evaluation of indoor spaces, thereby contributing to the overall well-being of their occupants.

## 1 Introduction

Restorative environments refer to places that promote the recovery of psychological and physiological processes elicited by specific environmental features and configurations (Hartig, 2004; Kaplan and Kaplan, 1989), which can be leveraged to improve individual and collective well-being (Miola and Pazzaglia, 2025; Pazzaglia and Tizi, 2022; Reyes-Riveros et al., 2021). The beneficial effects of restorative environments are grounded in two important theoretical frameworks: Stress Reduction Theory (SRT; Ulrich, 1983) and Attention Restoration Theory (ART; Kaplan and Kaplan, 1989). According to SRT, exposure to natural environments leads to a reduction in sympathetic nervous system activity and an increase in parasympathetic activity, resulting in decreased psychophysiological stress and a positive impact on mood, such as a reduction in negative emotions and an increase in positive ones. ART, on the other hand, posits that spending time in environments with certain restorative features can restore cognitive resources (e.g., voluntary attention) that are depleted during everyday tasks (Kaplan, 1995). Natural environments, as opposed to urban environments, are considered to possess the higher restorative qualities (Menardo et al., 2021). These qualities include fascination, which spontaneously captures interest and attention; being away, which provides a sense of escape from daily routines and introduces a change of experience; compatibility, reflecting the alignment between environmental features and individuals' needs, goals, and preferences; and extent, which refers to the coherence and broad scope of environmental elements. Together, these attributes have been shown to positively influence people's voluntary attention (Kaplan and Kaplan, 1989; Neilson et al., 2019; Ulrich, 1983). Besides restorativeness, our preference for natural settings is grounded in the concept of “biophilia” (Wilson, 1984), described as a biologically determined human predisposition to seek contact with nature and all living things. However, despite the well-documented benefits of nature, increasing urbanization (United Nations, 2018) has significantly reduced opportunities to access green spaces and other restorative environments (Van Den Berg et al., 2010). People now spend 90% of their time within built environments, resulting in a growing disconnection from the natural world (Kellert and Calabrese, 2015). One possible solution to this problem lies in a design approach called biophilic design (Kellert et al., 2008). This approach, part of the broader framework of restorative design, is based on biophilia theory and involves integrating natural elements into built environments (Kellert et al., 2008). Kellert proposes five principles to guide biophilic design. The first and the most relevant for this study involves the use of natural materials and the inclusion of natural elements (e.g., green plants) within a space; the second involves employing shapes that characterize natural elements (e.g., fractals, botanical patterns, spirals) within the design; the third refers to the inclusion of patterns and processes typically found in nature, such as sensory variability or hierarchical organization of elements; the fourth principle focuses on creating a connection between the built space and the surrounding environment, aligning with the specific habitat; and finally, the fifth principle addresses the relationship between humans and nature throughout evolution, aimed at evoking a sense of refuge and protection. The literature provides several examples of restorative design in settings such as schools, hospitals, and workplaces (e.g., Blaschke et al., 2017; Felgueiras et al., 2022; Lee and Yoon, 2023), whereas only a few studies focus on restorative and biophilic design of residential environments (Kim et al., 2010; Lim et al., 2009). Yet, of all the built environments, the home is the place where most of the population conducts nearly all daily activities, and the need to conceive of the domestic environment as more than just a physical space has been amplified by the COVID-19 pandemic, which required many individuals to remain at home throughout the day due to lockdowns (Fornara et al., 2022). The home, in fact, seems to reflect the identity and values of its occupants and is essential for the social and psychological well-being of its occupants (D'Alessandro et al., 2020; Gosling et al., 2002), as spatial adequacy is a factor to consider in stress reduction (Fornara et al., 2022). Environmental features, related to environmental satisfaction, have been studied primarily on a large scale (e.g., residential neighborhood satisfaction; Aragonés et al., 2017; Bonaiuto et al., 2015; Campagna, 2016) whereas the spatial characteristics of indoor environments (e.g., homes) have been less explored. Biophilic design has demonstrated various beneficial effects on individuals' physical and psychological health (Gillis and Gatersleben, 2015). In general, the inclusion of plants in indoor environments is associated with increased positive emotions, reduced negative emotions and relief from physical discomfort (Han and Ruan, 2019). Moreover, plants can contribute to general health promotion by lowering blood pressure, reducing stress levels, and improving indoor environmental quality (IEQ) (Yeo, 2020; Han and Ruan, 2019). A recent literature review highlighted that incorporating plants in indoor environments (mostly offices, schools, and hospitals) positively impacts perceptions of these spaces compared to plant-free environments (Han and Ruan, 2019). For example, rooms with plants are perceived as more comfortable, aesthetically pleasing and are associated with fewer negative emotions (e.g., pressure, anxiety, and fatigue), greater positive emotions (e.g., happiness, pleasantness, relaxation), and perceived restorativeness compared to rooms without plants (Evensen et al., 2017; Han and Hung, 2011; Hung and Han, 2010; Khan et al., 2005; Kim and Mattson, 2002; Yao et al., 2018). Most of the studies have analyzed potted plants, which are considered typical indoor plants (Van den Bogerd et al., 2021). Nonetheless, several types of indoor greenery exist, such as mobile plant dividers, permanent flower beds, or green walls and other solutions based on the integration of plants into furnishing elements. The integration of greenery into furnishings aims to provide practical benefits through technical design choices, such as a better use of space and simplification of the ongoing maintenance of plants, and architectural design is increasingly giving rise to innovative ideas and solutions proposed by architects and designers. Yet, these often remain subjective choices, influenced by the individual sensitivity of the designer or client, and their effects are rarely evaluated through scientific research. Therefore, studies specifically focused on these alternative design solutions remain limited and mainly centered on technical issues (Gunawardena and Steemers, 2019).

Environment evaluations are also influenced by individual characteristics and preferences. In fact, as claimed by Barbiero and Berto (2021), while the fascination and affiliation with nature have deep evolutionary roots, individual preferences for natural environments are also shaped by cognitive, affective, and cultural factors. Responses to nature depend not only on environmental features but also on the personal meanings, experiences, and emotions individuals attach to them. Personality traits, personal inclinations, and prior exposure influence how strongly one feels connected to, prefers, and benefits from natural settings. Biophilic tendencies require learning and contact with nature to be consolidated, and the interaction between innate predispositions and individual differences can determine the restorative value and appeal of natural or biophilic environments.

For example, the positive impact of nature has been linked to individuals' levels of connectedness to nature defined as the emotional and experiential bond between the self and the natural world. Research has shown that higher levels of connectedness to nature are associated with greater perceptions of environmental restorativeness (Berto and Barbiero, 2017). Another study (Sella et al., 2023) investigates personality factors showing a positive correlation between the extraversion personality traits and the perceived restorativeness of natural environments. Moreover, a preference for natural over built environments was positively associated with feelings of fascination and a sense of being away from everyday life (Sella et al., 2023). However, there is still limited evidence on the relationship between personality traits, nature connectedness, and the evaluation of biophilic spaces. Such individual differences may modulate the perception and appraisal of built environments, suggesting that specific design solutions, such as the integration of greenery, could be experienced in systematically different ways depending on the occupant's personality characteristics or level of nature connectedness. Considering these factors may provide a more nuanced understanding of the restorative effects of different domestic settings and furnishings.

The present study aims to investigate how domestic environments (such as living rooms and bedrooms) with or without the inclusion of different solutions of plants, are evaluated and perceived. Moreover, it explores whether these evaluations vary according to the type of greenery used in the interior design and whether certain individual characteristics may influence such evaluations.

To achieve these aims, we created virtual images of living rooms and bedrooms with different greenery conditions: integrated greenery, involved the integration of plants into furniture; potted greenery, included the placement of potted plants within the room; without greenery, serving as a control, excluded the presence of plants. Moreover, different variations of integrated greenery were devised: “Non-climbing integrated greenery” including plants incorporated into furniture modules, and “Climbing integrated greenery” including climbing plants supported by specific structures and furniture. This study represents the first experimental investigation to compare interior design approaches that incorporate plants integrated into furniture, while keeping its elements unchanged. The concept of integrating greenery into furniture aims to provide practical benefits through thoughtful technical design choices, such as simplifying both the purchasing process and the ongoing maintenance of plants. Given that most studies focus on the use of potted plants, our aim was to assess the impact of this innovative design approach compared to the well-documented positive effects of greenery.

The psychological effects of the conditions were examined in terms of perceived restorativeness, aesthetic appraisal, and affective qualities of the places.

According to past research conducted in public spaces (e.g., offices, schools, health settings; Han and Ruan, 2019) we expected that, compared to the plant-free, the plant-present conditions obtained higher scores in restorativeness, esthetic appraisal, positive affective qualities and lower scores in negative affective qualities. We were also interested in exploring whether integrated greenery (differing in climbing and non-climbing) obtained the same or superior evaluations as potted plants, in bedrooms and living rooms.

Finally, we seek to understand whether aesthetic judgments, assessments of the affective qualities and perceived restorativeness of spaces, were related to individual differences. In the context of biophilic design, individual differences and preferences have been less investigated, therefore we adopted an exploratory approach, examining individual differences in personality traits, inclusion of nature in the self, and preferences for natural vs. built environments.

In summary, previous literature on biophilic indoor environments and indoor plants has predominantly focused on schools, workplace settings and healthcare environments. However, fewer investigations have explored domestic spaces such as living rooms and bedrooms, making this an innovative aspect of the present study. By including these types of domestic environment, we address an important gap in the research. Furthermore, previous studies have generally examined indoor plants without distinguishing between different types of greenery or methods of integration. In contrast, our study examines multiple green conditions. This approach provides a more comprehensive understanding of how specific types of vegetation and their integration into interiors influence perceptions and evaluations of these spaces, also considering individual differences.

## 2 Materials and methods

### 2.1 Participants

The study involved a total of 331 participants. After processing, 304 questionnaires were considered valid for analysis, with 27 discarded due to incompleteness. The sample was randomly divided into two groups. The first group (Non-climbing integrated greenery) was composed of 152 participants (71 female, 79 male, 2 non-binary) with an average age of 32.38 years (SD = 9.59). The second group (Climbing integrated greenery) was composed of 152 participants (75 female, 74 male, 3 non-binary) with an average age of 35.58 years (SD = 11.05) (see Table 1 for additional information about age and gender). Each group was exposed to three conditions: potted greenery, greenery integrated into furniture (according to group condition), without greenery. The study was approved by the local ethical committee for Psychological Research at the University of Padova (protocol No. 570-b), and all participants gave informed consent.

**Table 1:** Frequency distribution of demographic information for the two groups.

		**Non-climbing greenery**	**Climbing greenery**
	**Frequency**	**Frequency**
Gender	Female	71	75
	Male	79	74
	Non-binary	2	3
	Total	152	152
Age	18–30	80	65
	31–40	43	45
	41–50	21	19
	51–60	6	19
	61–65	2	4
	Total	152	152

A posteriori power analysis was conducted to assess whether the study had sufficient power to detect a small-to-moderate effect size (f^2^ = 0.07) in a multiple regression model with 13 predictors. Using the pwr package in R, the analysis indicated that with 300 degrees of freedom, an alpha level of 0.05, the achieved power was 0.87. This suggests that the study had adequate power to detect small-to-moderate effects.

### 2.2 Instruments and materials

#### 2.2.1 Individual characteristics

##### 2.2.1.1 Demographic information

Demographic information such as gender, age, education level, income, and place of residence was collected through a specifically designed questionnaire.

##### 2.2.1.2 Big five inventory scale (BFI-10)

This 10-item questionnaire (Rammstedt and John, 2007) measures personality traits using a 5-point Likert scale (from 1 = strongly disagree to 5 = totally agree). For this study, we used the Italian version (Guido et al., 2015). It includes five subscales: “Conscientiousness”, “Openness”, “Agreeableness”, “Emotional stability”, and “Extraversion”. The dependent variables were the subscale scores. An example item is “I perceive myself as a person who is relaxed, copes well with stress”. The internal consistency of the Big Five subscales ranged as follows: Extraversion: α = 0.66; Agreeableness: α = 0.49; Conscientiousness: α = 0.39; Emotional Stability: α = 0.79; Openness: α = 0.53.

##### 2.2.1.3 Preference for nature questionnaire

This 10-item questionnaire (McMahan and Josh, 2017) measures the preference for natural environments over built ones using an 8-point Likert scale (from 1 = absolutely false to 8 = absolutely true). For this study, we used an Italian translation. The independent variable was the total score, with a higher score indicating greater preference for natural environments (over built ones). An example item is “I enjoy being in nature more than being in cities or urban areas”. The internal consistency of the PNQ was good (Cronbach's α = 0.90).

##### 2.2.1.4 Inclusion of nature in self

This 1-item questionnaire (Schultz, 2001) measures the extent to which participants felt connected to nature choosing one of the seven images presented using a 7-point Likert scale (from 1 = not at all connected with the natural world to 7 = completely connected with the natural world).

#### 2.2.2 Environmental perception

##### 2.2.2.1 Russell's model on affective qualities of places

This is an Italian instrument (Perugini et al., 2002) inspired by Russell's Circumplex Model (Russell and Lanius, 1984) that assesses the affective characteristics of places through a list of adjectives, organized according to a model based on 4 dimensions: “Relaxing-Stressful”, “Enthusiastic-Depressing”, “Pleasant-Unpleasant” and “Stimulating-Overwhelming/Noxious.” The questionnaire is made by a list of 48 adjectives, but for this study we selected only 6 adjectives for the affective quality of places (“restful”, “inviting”, “lively”, “chaotic”, “oppressive”, “monotonous”) and 1 adjective for the aesthetic judgment (“beautiful”). Participants are asked to express their degree of agreement with the adjectives that express their experience in the environment, on a 7-point Likert scale (from 1 = not at all suitable to 7 = completely suitable). The internal consistency of the affective qualities of Russell's model was good for positive adjectives (Cronbach's α = 0.91) and modest for negative adjectives (Cronbach's α = 0.51).

##### 2.2.2.2 Perceived restorativeness scale (PRS-5)

This 5-item questionnaire (Berto, 2005) assesses the perceived restorativeness of an environment through its beneficial/restorative properties, based on ART (Kaplan, 1995). For this study, we used only 4 items that corresponded to the 4 subscales: Being Away, Fascination, Coherence, and Compatibility. The dependent variables were the subscale scores and the total score. An example item is “This place contains various elements that spontaneously capture my attention”. The internal consistency of the short version of PRS was good (Cronbach's α =.86).

#### 2.2.3 Virtual images of real indoor environments

**Figure 1:**
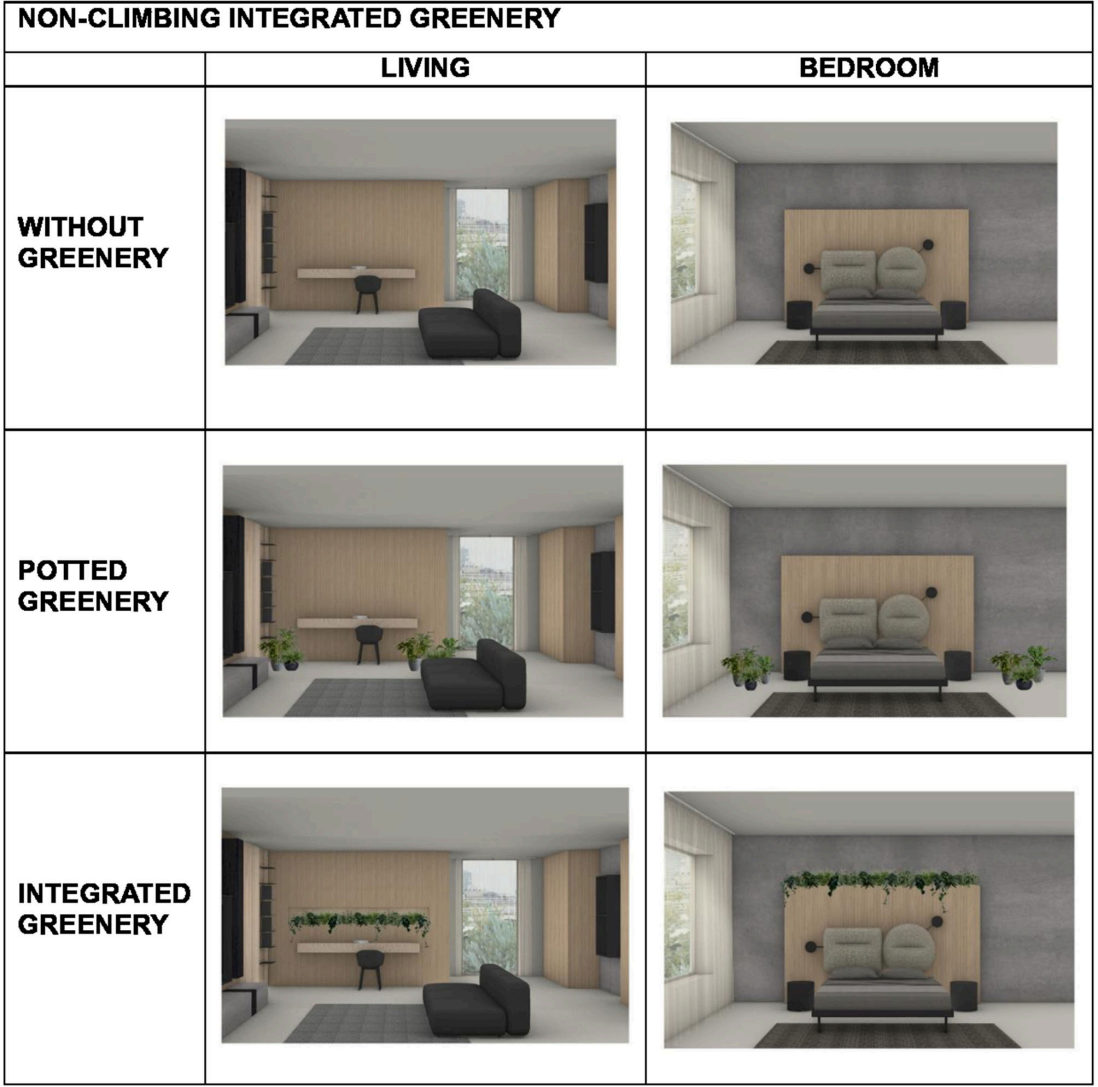
Examples of experimental images for the “Non-climbing integrated greenery” condition.

**Figure 2:**
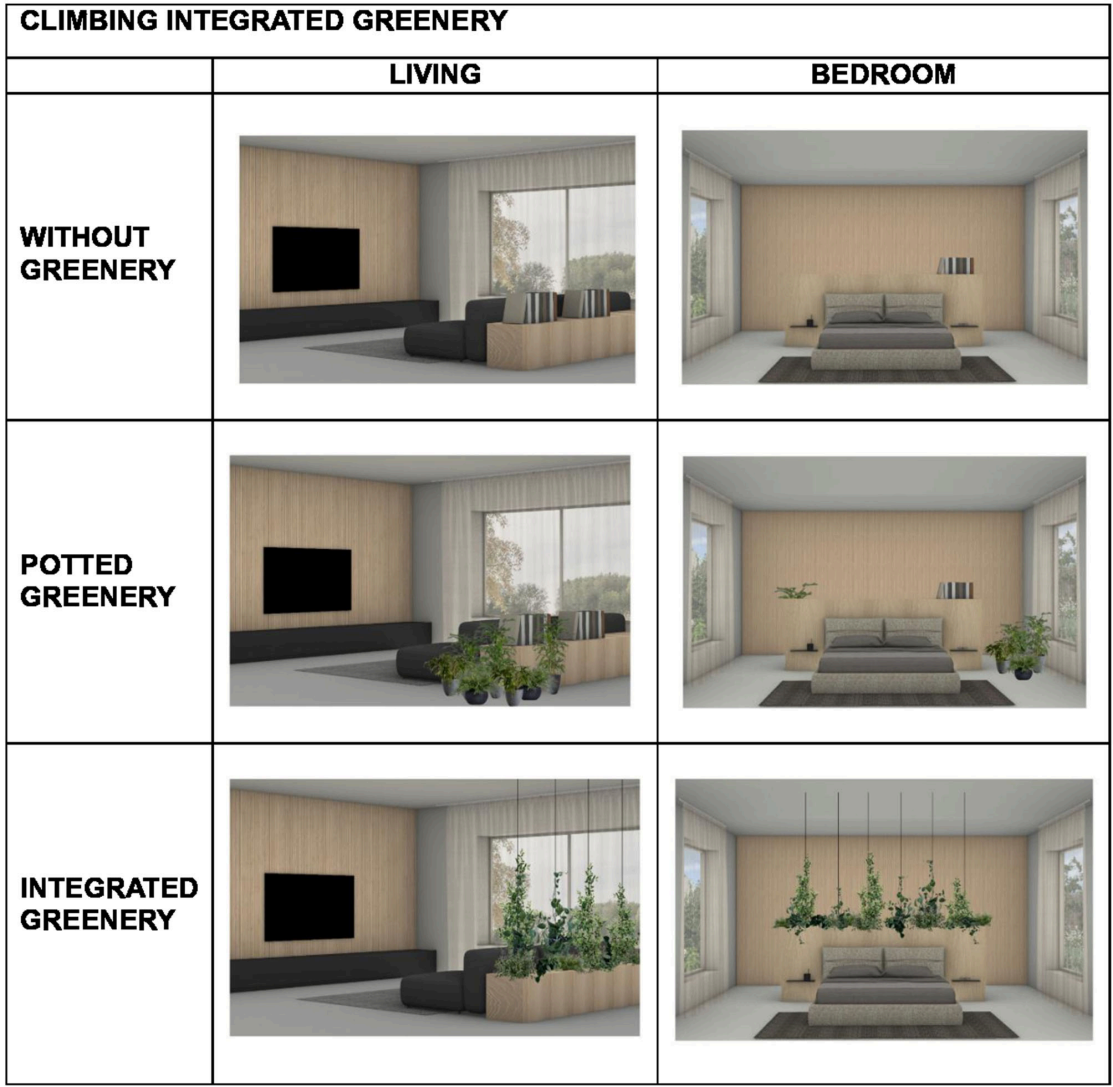
Examples of experimental images for the “Climbing integrated greenery” condition.

According to the between-subject condition (Climbing integrated greenery vs. Non-climbing integrated greenery) three living rooms and three bedrooms were designed for each condition. Each environment (i.e., each room) was created in three different versions: without greenery, with potted greenery and with greenery integrated into the furniture. Within each condition, the free versions were created by modifying only the type of greenery, while keeping all other architectural features constant (e.g., furniture, windows, materials, and colors; see Figures 1, 2).

All environments were digitally rendered using Rhinoceros, a 3D modeling software, to ensure consistency and control over visual variables. Figures 1, 2 show examples of the different types of environments, divided according to the two conditions between-subjects. In total, 36 images were created, comprising 12 environments presented across the three greenery conditions.

### 2.3 Procedure

Participants were recruited through the Prolific platform and randomly assigned to one of two conditions: Non-climbing integrated greenery vs. Climbing integrated greenery. The participants completed an online questionnaire via the Qualtrics platform, which took approximately 20 min. The first part of the online questionnaire collected demographic information and assessed personality traits, preference for urban or natural environments, and connection to nature. It lasted approximately 7 min. In the second part, each participant was exposed to a total of six images: one living room and one bedroom for each of the three greenery conditions (without greenery, potted greenery, and integrated greenery—according to the between-subject condition). Each image remained visible on the screen while the participants were asked to evaluate the affective qualities and the perceived restoration of the space. Participants could proceed to the next image at their own pace, allowing them adequate time for observation and evaluation. The order of image presentation was randomly assigned to the participants. This part lasted approximately 13 min.

### 2.4 Statistical analysis

Statistical analyses were conducted using R studio. First, we computed descriptive statistics for all variables (see Tables 2, 3). Then, we applied mixed-effects linear models to examine the relationships between individual differences (personality traits and preferences for natural and built environments), greenery conditions, room type, and both perceived restorativeness and affective qualities. Specifically, we explored how the predictors: Big Five personality traits, PNQ, room type, greenery condition (potted greenery, without greenery, integrated greenery), variation (furniture with non-climbing integrated greenery vs. furniture with climbing integrated greenery), related to the dependent variables: (1) PRS and its subscales and (2) affective qualities of the environment, based on Russell's model.

**Table 2:** Means and standard deviations of dependent variables divided by type of green.

**Dependent**	**Potted greenery**		**Without greenery**		**Integrated greenery**	
**variables**	**Mean**	**SD**	**Mean**	**SD**	**Mean**	**SD**
Restorativeness	18.14	5.02	16.16	5.54	18.17	5.12
Being away	4.67	1.52	4.16	1.73	4.60	1.53
Fascination	4.24	1.60	3.45	1.72	4.42	1.58
Coherence	5.05	1.45	4.93	1.55	4.93	1.50
Compatibility	4.17	1.65	3.61	1.75	4.20	1.73
Restful	4.70	1.36	4.20	1.54	4.70	1.47
Beautiful	4.42	1.44	3.89	1.62	4.49	1.52
Inviting	4.37	1.42	3.73	1.58	4.38	1.50
Lively	3.15	1.51	2.49	1.39	3.28	1.57
Chaotic	2.00	1.28	1.93	1.31	2.07	1.36
Oppressing	2.56	1.52	3.07	1.72	2.58	1.54
Monotonous	4.04	1.68	4.73	1.73	3.93	1.72

**Table 3:** Means and standard deviations of dependent variable divided by type of room (bedroom vs. living).

**Dependent**	**Bedroom**		**Living**	
**variables**	**Mean**	**SD**	**Mean**	**SD**
Restorativeness	17.35	5.26	17.63	5.37
Being away	4.43	1.61	4.52	1.61
Fascination	4.07	1.68	4.01	1.69
Coherence	4.87	1.54	1.69	5.06
Compatibility	3.97	1.73	4.02	1.73
Restful	4.58	1.48	4.49	1.47
Beautiful	4.21	1.56	4.32	1.54
Inviting	4.09	1.54	4.23	1.52
Lively	2.91	1.50	3.04	1.56
Chaotic	2.05	1.34	1.95	1.29
Oppressing	2.79	1.62	2.69	1.60
Monotonous	4.28	1.73	4.19	1.76

Predictors were entered into the models in the following order: Big Five traits, PNQ, room type, greenery conditions and variation. Additionally, we examined interactions between the condition of the greenery and the type of room. All models were estimated using Linear Mixed-Effects Regression Models (LMER) with the *lme4* package (Bates et al., 2015). Post hoc analyses were performed using pairwise contrast tests from the *emmeans* package (Lenth, 2021) when necessary, with Bonferroni correction applied for multiple comparisons. To account for individual differences, participants were included as a random factor, as they completed multiple questionnaire responses. Before running the models, continuous predictors were standardized.

## 3 Results

Tables 2, 3 showed descriptive statistics of variables divided by type of greenery and type of room. Tables showing correlational analysis and linear mixed models are reported in Supplementary Tables S1–S18, Means' distributions of restorativeness and affective qualities of rooms are shown in Supplementary Figures S1–S11, and a visual summary of the principal results is presented in Table 4.

**Table 4:** Visual summary of the results.

Individual differences	The trait of Extraversion is associated with more positive evaluations of the aesthetic qualities of spaces and with a lower perception of chaos. The trait of Agreeableness is associated with a higher perception of liveliness and a lower perception of oppression. The trait of Emotional Stability is associated with a higher perception of liveliness. The trait of Conscientiousness is associated with a lower perception of chaos. The trait of Openness to Experience is associated with a lower perception of chaos but also with a lower perception of liveliness. The Inclusion of Nature in the Self and the Preference for Natural over Built environments is associated with a lower perception of chaos.
Climbing integrated greenery in indoor environments	**Furniture with climbing integrated greenery** was rated significantly higher in terms of restful and beautiful, less oppressive characteristics than non-climbing integrated greenery.
Type of rooms	**Living rooms** generally benefited more from greenery, especially in terms of coherence, compatibility, and affective qualities. **Bedrooms** appeared to be more sensitive to the type and integration of greenery, with non-climbing integrated designs sometimes increasing negative perceptions (e.g., chaotic, monotonous).
Greenery in indoor environments	**Potted greenery** had an impact on general restorativeness as rooms without greenery consistently showed lower scores across restorativeness and its dimensions (being away, fascination, and compatibility). Moreover, potted greenery was associated with higher coherence compared to both no greenery and integrated greenery. **Integrated greenery** significantly increased fascination compared to both no greenery and potted greenery.

### 3.1 Individual differences

Regarding personality traits, the correlational analyses revealed a weak positive relationship between **Extraversion** and aesthetic judgment (*r* = 0.122, *p* < 0.05), suggesting that higher levels of Extraversion are associated with more positive evaluations of the aesthetic quality of the environments. A weak positive association also emerged between the traits of **Agreeableness** and **Emotional Stability** and the perception of liveliness (*r* = 0.113, *p* < 0.05; *r* = 0.120, *p* < 0.05, respectively), indicating that individuals scoring higher on these traits tended to rate the environments as more lively.

In addition, weak negative correlations were found between **Conscientiousness**, **Openness to Experience**, and **Extraversion** and the perception of chaos (*r* = −0.136, *p* < .05; *r* = −0.236, *p* < 0.001; *r* = −0.113, *p* < 0.05, respectively), suggesting that higher levels of these traits are associated with lower perception of environmental disorder.

Further weak negative relationships was found between **Agreeableness** and the perception of oppressiveness (*r* = −0.131, *p* < 0.05) and between **Openness to Experience** and the perception of liveliness (*r* = – 0.174, *p* < 0.01), indicating that individuals with higher levels of Agreeableness perceived the environments as less oppressive, while individuals higher in Openness to Experience perceived the environments as less lively.

Regarding nature connectedness, a weak negative correlation emerged between both the **Inclusion of Nature in the Self** and **Preference for Natural over Built Environments** and the perception of chaos (*r* = −0.161, *p* < 0.01; *r* = −0.179, *p* < 0.01, respectively). This suggests that individuals with stronger nature connectedness and a greater preference for natural environments tend to perceive the spaces as less chaotic. All reported correlations and their corresponding statistical values are presented in detail in Supplementary Table S1.

### 3.2 Environmental restorativeness

From linear mixed models, the results of the total score of **restorativeness** questionnaire showed an effect of the type of greenery (β = −0.38, *p* < 0.001) suggesting that no greenery is associated with a lower restorativeness score compared to the reference condition with potted greenery. The predictors explained 4% of the variance, 46% including random effects (see Supplementary Table S2).

Regarding **being away**, type of greenery showed an effect (β = −0.31, *p* < 0.001), suggesting that no greenery is associated with a lower score on being away compared to potted greenery. The predictors explained 3% of the variance, 44% including random effects (see Supplementary Table S3).

As before, the type of greenery had a significant effect on **fascination** (β = −0.47, *p* < 0.001; β = 0.20, *p* = 0.001) suggesting that no greenery is associated with a lower score than potted greenery, moreover the latter is associated with a significantly lower score on fascination compared to integrated greenery. An interaction between the type of room and the type of greenery emerged (β = −0.20, *p* = 0.03) (see Supplementary Table S4). Post-hoc analyses showed that Bedroom with integrated greenery has higher fascination scores than Bedroom without greenery and potted greenery. Moreover, Living room with integrated greenery showed higher scores that Living room without greenery. The predictors explained 7% of the variance, 39% including random effects (see Supplementary Table S5).

Concerning **coherence**, the type of greenery emerged as significant predictor (β = −0.12, *p* = 0.04; β = −0.17, *p* = 0.004) suggesting that no greenery, and integrated greenery are associated with a significantly lower score on coherence compared to potted greenery. Moreover, it emerged an interaction between type of room and the type of greenery (β = 0.19, *p* = 0.03) (see Supplementary Table S6). Post-hoc analyses revealed that Living rooms with potted greenery showed higher scores than bedroom with integrated greenery and Living room with integrated greenery showed higher scores than bedroom with integrated greenery. The predictors explained 2% of variance, 40% including random effects (see Supplementary Table S7).

Finally, for **compatibility**, the type of greenery had a main significant effect (β = −0.30, *p* < 0.001) suggesting that the condition without greenery is associated with lower scores on compatibility compared to potted greenery. Moreover, the type of room emerged as significant (β = 0.15, *p* = 0.04) showing that the living room space compared to bedroom showed higher levels of compatibility. The predictors explained 4% of the variance, 42% including random effects (see Supplementary Table S8).

### 3.3 Affective qualities of rooms

Among the affective qualities, for the **restful** factor the type of greenery emerged as significant (β = −0.29, *p* < 0.001) suggesting that rooms without greenery are associated with a significantly lower scores on restful compared to potted greenery. Moreover, a significant effect of variation of integrated greenery emerged (β = −0.20, *p* = 0.02) indicating that furniture with non-climbing integrated greenery has lower scores on restful characteristics than furniture with climbing integrated greenery (see Supplementary Table S9). The predictors explained 4% of the variance, 44% including random effects.

As for **beautiful** characteristics, a main effect of the type of greenery emerged (β = −0.35, *p* < 0.001) suggesting that the rooms without greenery are associated with lower scores compared to potted greenery. Moreover, a significant effect of variation of integrated greenery emerged (β = −0.20, *p* = 0.02) suggesting that furniture with non-climbing integrated greenery showed lower beautiful scores compared to furniture with climbing integrated greenery (see Supplementary Table S10). The predictors explained 5% of the variance, 46% including random effects.

As for **inviting** characteristics, a main effect of type of room emerged (β = −0.02, *p* < 0.001) suggesting that the living room space is associated with a significantly higher scores on inviting characteristics compared to bedroom. Moreover, the type of greenery had a main significant and negative effect (β = −0.03, *p* < 0.001): no greenery is associated with lower scores compared to potted greenery. Finally, an interaction between type of room and variation of integrated greenery emerged (β = −0.01, *p* = 0.03) (see Supplementary Table S11). Post-hoc analyses showed that within the climbing integrated greenery condition Living room showed higher scores than bedrooms on inviting characteristics. The predictors explained 5% of the variance, 42% including random effects (see Supplementary Table S12).

Regarding **lively** characteristics, the type of room had an effect (β = 0.18, *p* < 0.009) suggesting that the living rooms are associated with higher scores compared to bedrooms. Moreover, the type of greenery emerged (β = −0.40, *p* < 0.001) suggesting that no greenery is associated with lower scores on lively characteristics compared to potted greenery.

Finally, individual differences of openness had a main significant effect (β = −0.11, *p* = 0.003) suggesting that higher openness is associated with lower levels of perceived lively characteristics (see Supplementary Table S13). The predictors explained 9% of the variance, 45% including random effects.

As for **chaotic** characteristics, the type of greenery had a main effect (β = 0.14, *p* = 0.03) indicating that integrated greenery has higher score on chaotic characteristics compared to potted greenery. Moreover, an interaction between type of room and variation of integrated greenery emerged (β = −0.22, *p* = 0.004) (see Supplementary Table S14). Post-hoc analyses showed that within the non-climbing integrated greenery condition Bedrooms showed higher scores on chaotic than living rooms.

Finally, individual characteristics of openness and preferences for natural environment had a main significant effect on chaotic characteristics respectively (β = −0.13, *p* = 0.0001; β = −0.08, *p* = 0.02) suggesting that higher scores on openness and preference for natural environment is associated with lower perception of chaotic characteristics (see Supplementary Table S15). The predictors explained 5% of the variance, 31% including random effects.

A significant effect of variation of integrated greenery emerged on **oppressive** characteristic (β = 0.23, *p* = 0.006) suggesting that furniture with non-climbing integrated greenery has higher scores that furniture with climbing integrated greenery. Moreover, the type of greenery had a main significant effect (β = 0.37, *p* < 0.001) suggesting that no greenery is associated with higher scores than potted greenery (see Supplementary Table S16). The predictors explained 5% of the variance, 41% including random effects.

Finally, for **monotonous** characteristics a main effect of the type of room (β = −0.21, *p* = 0.004) indicates that living rooms have lower scores compared to bedroom spaces. Moreover, the type of greenery emerged (β = 0.36, *p* < 0.001; β = −0.13, *p* = 0.04) suggesting that no greenery, and integrated greenery are associated respectively with a significantly higher and lower scores on monotonous characteristics compared to potted greenery. Finally, an interaction between the type of room and variation of integrated greenery emerged (β = 0.19, *p* = 0.01) (see Supplementary Table S17). Post-hoc analyses revealed that within the climbing integrated greenery condition, Bedrooms showed higher scores of monotonous characteristics than Living rooms (see Supplementary Table S18). The predictors explained 6% of the variance, 36% including random effects.

## 4 Discussion

Since natural environments are the most restorative, a design approach known as biophilic design aims to introduce natural elements into built environments to elicit similar beneficial effects (Kellert et al., 2008). The review by Han and Ruan (2019) highlighted that many studies have focused on investigating the effects of biophilic design strategies in public spaces (e.g., schools, hospitals, offices; Han, 2018; Park and Mattson, 2008; Yao et al., 2018) often overlooking the importance of domestic environments. Given the amount of time people spend at home, investigating the role of biophilic design in residential settings is especially relevant. In this regard, the main aim of this study was to examine how specific domestic spaces (living rooms, bedrooms) are perceived and evaluated (in terms of perceived restorativeness, aesthetic judgment, and affective qualities) with or without the inclusion of plants, to fill the gap in the literature. Furthermore, given that most research on indoor greenery has focused on potted plants (Gunawardena and Steemers, 2019) rather than other forms of vegetation, we create ad hoc environments where greenery was integrated into the furniture in two different ways to explore potential differences in perception and judgment; therefore we kept constant characteristics of the interior rooms such as furniture, wall color, materials, window positions and created variations by only changing the type of greenery inside with potted greenery, integrated greenery in the furniture and no greenery at all. Additionally, due to the limited research on domestic environments, we investigated whether room type (Living room vs. Bedroom) influenced these evaluations. Finally, our interest was to explore whether individual differences such as personality traits or preference for natural environments over built ones could have a role on aesthetic judgments, perceived restorativeness, and the evaluation of affective qualities of the domestic spaces.

Regarding the effect of indoor greenery, in line with previous literature, our findings indicate that rooms containing potted plants elicit a greater perception of restorativeness than rooms without greenery (Han and Hung, 2011; Hung and Han, 2010). Specifically, rooms with potted plants also generate a stronger sense of escape and detachment from everyday life, and a heightened sense of fascination, coherence, and compatibility between individuals' needs and environmental characteristics. Moreover, in line with the studies by Evensen et al. (2017) and Khan et al. (2005), rooms containing potted plants were rated more aesthetically pleasing than rooms without greenery. Additional findings indicate that such rooms are also perceived as more relaxing, inviting, and lively, while being perceived as less oppressive and monotonous. Interestingly, incorporating greenery directly into the furniture (as opposed to placing potted plants freely in the room) increased perceived fascination and reduced monotony. Moreover, it also decreased perceived coherence and made the environment feel more chaotic compared to rooms with potted plants, likely due to the lower familiarity with this type of greenery in indoor environments.

The perception of these effects also varied by room type. Bedrooms with integrated greenery were perceived as more fascinating than bedrooms without greenery or with potted greenery, but they were judged to be less coherent compared to living rooms with potted or integrated greenery. Additionally, living rooms with integrated greenery were rated as more fascinating than living rooms without greenery.

When evaluating the effects of different types of greenery integration, the results showed that rooms with climbing greenery integrated into the furniture were rated as more aesthetically pleasing, more relaxing, and less oppressive than those with non-climbing green integration. Considering the variations of integrated greenery, Climbing integrated greenery in living rooms made them appear more inviting and less monotonous compared to bedrooms, whereas Non-climbing integrated greenery made bedrooms seem more chaotic than living rooms. Contrary to our expectations, no significant differences in perceived restorativeness emerged between the climbing and non-climbing settings. These two novel design solutions are likely to elicit a greater sense of fascination compared to the more traditional conditions of potted plants or the absence of greenery. However, the difference between them may not be strong enough to produce a differentiated perception of restorativeness. In contrast, affective quality judgments appear to vary depending on the specific room being evaluated.

Regarding individual differences, we found weak correlations between certain personality traits, inclusion of nature in the self, preference for natural environments, and the affective evaluations of the spaces (see Supplementary Table S1). Specifically, higher levels of Extraversion were associated with more positive aesthetic judgments of environments in general. Greater levels of Agreeableness and Emotional Stability were related to higher ratings of liveliness, whereas higher levels of Conscientiousness, Openness and Extraversion were associated with lower perception of disorder. Moreover, greater Agreeableness was linked to lower feelings of oppressiveness, while greater Openness to Experience was linked to lower feelings of liveliness. Regarding nature connectedness, both higher inclusion of nature in the self and a stronger preference for natural over built environments were associated with reduced perceptions of disorder in the evaluated spaces. Nonetheless, individual factors did not appear to play a substantial role in shaping people's perceptions of environments in terms of their perceived restorativeness and affective quality. This suggests that the presence of certain environmental features (such as presence of greenery) exerts a primary effect. However, it was important to control for these individual differences in the analysis. Specifically, only openness and preferences for natural environments emerged as relevant predictors, and results suggest that individuals who are open to experience and prefer natural environments over built ones tend to perceive spaces as less chaotic. In contrast, individuals who are open to experience tend to judge the environments as less lively.

Finally, our findings also suggest that the impact of greenery may vary depending on the type of room. This highlights the importance of considering that different domestic spaces may require distinct design solutions and although weak positive correlations emerged between certain personality traits and more favorable evaluations of the environments, personality traits do not appear to play a central role in shaping how green-enhanced spaces are perceived. This finding is particularly valuable, as it suggests that the design of such nature-integrated furnishings can follow a more standardized approach while still effectively promoting the well-being of a broad and diverse range of users; nevertheless, more research is needed to replicate and refine these findings, especially considering the potential role of other psychological or contextual factors not captured in the current study.

## 5 Limitations

While the present study offers valuable insights into how different types of greenery are perceived in domestic environments, several limitations should be acknowledged.

First, our experimental design allows for a direct comparison among the three conditions (without greenery, potted greenery, and integrated greenery) within both the climbing and non-climbing scenarios, as identical environments were presented in a balanced manner across these conditions. However, it is important to note that direct comparisons between the climbing and non-climbing effects should be interpreted with caution, as different environments were used, potentially introducing confounding variables. Second, a limitation concerns the type of greenery examined and the rooms considered. Our study focused exclusively on traditional potted plants and a novel form of greenery integration with two variations, tested in living rooms and bedrooms. Future research should explore a wider range of integrated greenery solutions to better understand which are most effective. Moreover, exploring long-term effects on actual occupants (rather than relying solely on image-based evaluations) would deepen our understanding of how biophilic design affects well-being in everyday life. Furthermore, although we considered personality traits and connectedness to nature, the internal consistency of the BFI-10 subscales was relatively low. This is in part due to the general characteristics of the instrument itself as commonly reported in previous studies using this brief instrument (Biolcati et al., 2022; Nardozza et al., 2025). This limitation may have weakened the strength of the observed associations. While we found weak positive correlations between certain traits and environmental judgments, these results should be interpreted with caution, and future studies using more comprehensive personality measures are warranted.

## 6 Conclusions

In conclusion, the introduction of greenery into domestic environments has positive effects on perception and judgment of the indoor spaces. Given the importance of the home for the social and psychological well-being of its occupants, furnishings that elicit more positive evaluations and a greater sense of restorativeness may enhance the well-being of individuals who spend most of their time indoor, reinforcing the idea that even small design choices can significantly shape users' experience of space. Due to the limited research on the effects of different types of greenery in domestic environments, our study contributes to the existing literature on this topic, especially because we investigated distinct constructs such as perceived restorativeness and evaluations of affective qualities in order to understand which design solution has a more positive impact on psychological well-being, whereas most existing studies comparing different types of indoor greenery have focused primarily on outcomes related to air quality, thermal comfort or energy efficiency (Gunawardena and Steemers, 2019).

## Data Availability

The raw data supporting the conclusions of this article will be made available by the authors, without undue reservation.
