# Supplementary material for: Green design in living and bedroom spaces: exploring environmental restorativeness and affective qualities of spaces
Source: Front Psychol. 2025 Oct 31;16:1631417. doi: 10.3389/fpsyg.2025.1631417 (PMC12615197; doi:10.3389/fpsyg.2025.1631417)

**SUPPLEMENTARY MATERIAL for**

***Green Design in Living and Bedroom Spaces: Exploring Environmental Restorativeness and Affective Qualities of Spaces***

**Table S1.** Correlation matrix between individual factors and PRS-related variables. Statistically significant correlations are indicated with asterisks (**p* < .05, ***p* < .01, **p < .001).*

|  | *(1)* | *(2)* | *(3)* | *(4)* | *(5)* | *(6)* | *(7)* | *(8)* | *(9)* | *(10)* | *(11)* | *(12)* | *(13)* | *(14)* | *(15)* | *(16)* | *(17)* | *(18)* | *(19)* |
| --- | --- | --- | --- | --- | --- | --- | --- | --- | --- | --- | --- | --- | --- | --- | --- | --- | --- | --- | --- |
| *1. INS* |  |  |  |  |  |  |  |  |  |  |  |  |  |  |  |  |  |  |  |
| *2.PNQ* | 0.635^***^ |  |  |  |  |  |  |  |  |  |  |  |  |  |  |  |  |  |  |
| *3.BF_AGR* | 0.138^*^ | 0.167^**^ |  |  |  |  |  |  |  |  |  |  |  |  |  |  |  |  |  |
| *4. BF_CON* | 0.245^***^ | 0.217^***^ | 0.100 |  |  |  |  |  |  |  |  |  |  |  |  |  |  |  |  |
| *5. BF_ES* | 0.105 | 0.046 | 0.290^***^ | 0.218^***^ |  |  |  |  |  |  |  |  |  |  |  |  |  |  |  |
| *6. BF_OPE* | 0.147^*^ | 0.091 | -0.012 | 0.065 | -0.115^*^ |  |  |  |  |  |  |  |  |  |  |  |  |  |  |
| *7. BF_EXT* | 0.142^*^ | 0.034 | 0.188^***^ | 0.205^***^ | 0.182^**^ | 0.090 |  |  |  |  |  |  |  |  |  |  |  |  |  |
| *8. PRS_tot* | -0.010 | -0.056 | 0.015 | -0.001 | 0.030 | -0.023 | 0.038 |  |  |  |  |  |  |  |  |  |  |  |  |
| *9. PRS_BA* | -0.028 | -0.061 | 0.012 | -0.028 | 0.053 | -0.047 | 0.059 | 0.906^***^ |  |  |  |  |  |  |  |  |  |  |  |
| *10. PRS_FA* | 0.030 | -0.063 | -0.005 | -0.009 | -0.034 | -0.030 | -0.020 | 0.828^***^ | 0.642^***^ |  |  |  |  |  |  |  |  |  |  |
| *11. PRS_COH* | 0.017 | 0.030 | 0.064 | 0.071 | 0.036 | 0.090 | 0.075 | 0.722^***^ | 0.549^***^ | 0.442^***^ |  |  |  |  |  |  |  |  |  |
| *12. PRS_COM* | -0.047 | -0.088 | -0.014 | -0.031 | 0.044 | -0.080 | 0.017 | 0.907^***^ | 0.842^***^ | 0.703^***^ | 0.480^***^ |  |  |  |  |  |  |  |  |
| *13. relaxing* | 0.036 | -0.023 | 0.064 | 0.019 | 0.056 | -0.036 | 0.076 | 0.842^***^ | 0.879^***^ | 0.618^***^ | 0.550^***^ | 0.776^***^ |  |  |  |  |  |  |  |
| *14. beautiful* | 0.017 | -0.008 | 0.040 | 0.037 | 0.054 | -0.040 | 0.122^*^ | 0.832^***^ | 0.790^***^ | 0.672^***^ | 0.497^***^ | 0.825^***^ | 0.830^***^ |  |  |  |  |  |  |
| *15. inviting* | 0.038 | -0.042 | 0.069 | 0.013 | 0.082 | -0.082 | 0.056 | 0.825^***^ | 0.791^***^ | 0.695^***^ | 0.461^***^ | 0.813^***^ | 0.829^***^ | 0.836^***^ |  |  |  |  |  |
| *16. lively* | 0.042 | -0.036 | 0.113^*^ | 0.075 | 0.120^*^ | -0.174^**^ | 0.111 | 0.571^***^ | 0.513^***^ | 0.550^***^ | 0.207^***^ | 0.629^***^ | 0.553^***^ | 0.613^***^ | 0.693^***^ |  |  |  |  |
| *17. chaotic* | -0.161^**^ | -0.179^**^ | -0.106 | -0.136^*^ | -0.075 | -0.236^***^ | -0.113^*^ | -0.111 | -0.112 | 0.077 | -0.369^***^ | 0.007 | -0.127^*^ | -0.068 | 0.025 | 0.276^***^ |  |  |  |
| *18. oppressing* | 0.014 | -0.063 | -0.131^*^ | -0.101 | -0.087 | -0.018 | -0.079 | -0.453^***^ | -0.474^***^ | -0.313^***^ | -0.302^***^ | -0.429^***^ | -0.462^***^ | -0.443^***^ | -0.397^***^ | -0.102 | 0.384^***^ |  |  |
| *19. monotonous* | -0.036 | 0.024 | -0.066 | -0.078 | -0.072 | 0.102 | -0.035 | -0.433^***^ | -0.390^***^ | -0.478^***^ | -0.050 | -0.515^***^ | -0.377^***^ | -0.513^***^ | -0.528^***^ | -0.506^***^ | -0.028 | 0.430^***^ |  |
| *Computed correlation used pearson-method with listwise-deletion.* | | | | | | | | | | | | | | | | | | | |

***Note***: INS: Inclusion of Nature in Self; PNQ: Preference for Nature Questionnaire; BF_AGR: Agreeableness - Big Five dimension; BF_CON: Conscientiousness - Big Five dimension; BF_ES: Emotional stability - Big Five dimension; BF_OPE: Openness to experience - Big Five dimension; BF_EXT: Extraversion - Big Five dimension; PRS_TOT: Total score of the Perceived restorativeness (PRS-11); PRS_BA: Being away - PRS-11subscale; PRS_FA: Fascination - PRS-11subscale; PRS_COH: Coherence - PRS-11subscale; PRS_COM: Compatibility - PRS-11subscale; Relaxing: Russell's model dimension; Beautiful: Russell's model dimension; Inviting: Russell's model dimension; Lively: Russell's model dimension; Chaotic: Russell's model dimension; Oppressing: Russell's model dimension; Monotonous: Russell's model dimension.

**Table S2.** Mixed-effect linear model for Restorativeness

| *Restorativeness* | β | | 95%CI | *p* |
| --- | --- | --- | --- | --- |
| + BF_Agree | -0.008 | | [-0.07, 0.09] | 0.84 |
| + BF_Con | -0.001 | | [-0.08, 0.08] | 0.97 |
| + BF_Es | 0.019 | | [-0.06, 0.10] | 0.67 |
| + BF_Ext | 0.023 | | [-0.06, 0.10] | 0.58 |
| + BF_Ope | -0.009 | | [-0.09, 0.07] | 0.82 |
| + PNQ | | -0.041 | [-0.12, 0.04] | 0.33 |
| + type of room [living] | 0.108 | | [-0.02, 0.24] | 0.12 |
| + Variation [non-climbing] | -0.072 | | [-0.24, 0.10] | 0.42 |
| **+ type of greenery [without greenery]** | **-0.381** | | **[-0.49 -0.26]** | **<.001** |
| + type of greenery [integrated greenery] | 0.027 | | [-0.09, 0.14] | 0.65 |
| + type of room *type of greenery [without greenery] | 0.015 | | [-0.15, 0.18] | 0.86 |
| + type of room *type of greenery [integrated greenery] | -0.043 | | [-0.21, 0.12] | 0.61 |
| +type of room [living]* Variation | -0.093 | | [-0.22, 0.04] | 0.18 |

**Table S3.** Mixed-effect linear model for Being away

| *Being away* | β | | 95%CI | *p* |
| --- | --- | --- | --- | --- |
| + BF_Agree | -0.001 | | [-0.08, 0.08] | 0.98 |
| + BF_Con | -0.027 | | [-0.11, 0.05] | 0.52 |
| + BF_Es | 0.037 | | [-0.04, 0.12] | 0.39 |
| + BF_Ext | 0.043 | | [-0.03, 0.12] | 0.31 |
| + BF_Ope | -0.025 | | [-0.10, 0.05] | 0.55 |
| + PNQ | | -0.037 | [-0.12, 0.04] | 0.38 |
| + type of room [living] | 0.033 | | [-0.10, 0.17] | 0.63 |
| + Variation [non-climbing] | -0.106 | | [-0.27, 0.06] | 0.23 |
| **+ type of greenery [without greenery]** | **-0.311** | | **[-0.43, -0.19]** | **<.001** |
| + type of greenery [integrated greenery] | -0.073 | | [-0.19, 0.04] | 0.23 |
| + type of room *type of green [without greenery] | -0.004 | | [-0.17, 0.16] | 0.96 |
| + type of room *type of greenery [integrated greenery] | 0.069 | | [-0.10, 0.23] | 0.42 |
| +type of room[living]* Variation | 0.005 | | [-0.13, 0.14] | 0.94 |

**Table S4.** Mixed-effect linear model for Fascination

| *Fascination* | β | | 95%CI | *p* |
| --- | --- | --- | --- | --- |
| + BF_Agree | 0.012 | | [-0.06, 0.08] | 0.76 |
| + BF_Con | 0.009 | | [-0.06, 0.08] | 0.81 |
| + BF_Es | -0.020 | | [-0.09, 0.05] | 0.61 |
| + BF_Ext | -0.012 | | [-0.08, 0.06] | 0.76 |
| + BF_Ope | -0.013 | | [-0.08, 0.05] | 0.72 |
| + PNQ | | -0.041 | [-0.11, 0.03] | 0.28 |
| + type of room [living] | 0.066 | | [-0.07, 0.21] | 0.37 |
| + Variation [non-climbing] | -0.110 | | [-0.27, 0.05] | 0.19 |
| **+ type of greenery [without greenery]** | **-0.472** | | **[-0.59, -0.34]** | **<.001** |
| **+ type of greenery [integrated greenery]** | **0.209** | | **[-0.08, 0.33]** | **0.001** |
| + type of room *type of greenery [without greenery] | 0.011 | | [-0.16, 0.18] | 0.90 |
| **+ type of room *type of greenery [integrated greenery]** | **-0.202** | | **[-0.37, -0.02]** | **0.03** |
| +type of room [living]* Variation | -0.073 | | [-0.21, 0.07] | 0.32 |

**Table S5.** Post-hoc comparisons in Fascination.

| **Contrast** | **Estimate** | **SE** | **t-ratio** | **p-value** |
| --- | --- | --- | --- | --- |
| Bedroom with greenery - Living with greenery | -0.03 | 0.06 | -0.46 | 1.00 |
| **Bedroom with greenery - Bedroom without greenery** | **0.47** | **0.06** | **7.40** | **<.0001** |
| **Bedroom with greenery - Living without greenery** | **0.43** | **0.06** | **6.76** | **<.0001** |
| **Bedroom with greenery - Bedroom integrated greenery** | **-0.21** | **0.06** | **-3.29** | **0.02** |
| Bedroom with greenery - Living integrated greenery | -0.04 | 0.06 | -0.58 | 1.00 |
| **Living with greenery - Bedroom without greenery** | **0.50** | **0.06** | **7.85** | **<.0001** |
| **Living with greenery - Living without greenery** | **0.46** | **0.06** | **7.21** | **<.0001** |
| **Living with greenery - Bedroom integrated greenery** | **-0.18** | **0.06** | **-2.83** | **0.07** |
| Living with greenery - Living integrated greenery | -0.01 | 0.06 | -0.12 | 1.00 |
| Bedroom without greenery - Living without greenery | -0.04 | 0.06 | -0.64 | 1.00 |
| **Bedroom without greenery - Bedroom integrated greenery** | **-0.68** | **0.06** | **-10.68** | **<.0001** |
| **Bedroom without greenery - Living integrated greenery** | **-0.51** | **0.06** | **-7.97** | **<.0001** |
| **Living without greenery - Bedroom integrated greenery** | **-0.64** | **0.06** | **-10.04** | **<.0001** |
| **Living without greenery - Living integrated greenery** | **-0.47** | **0.06** | **-7.33** | **<.0001** |
| Bedroom integrated greenery - Living integrated greenery | 0.17 | 0.06 | 2.71 | 0.10 |

*Note: Significant results are marked with an asterisk (*p < 0.05).*

**Table S6.** Mixed-effect linear model for Coherence

| *Coherence* | β | | 95%CI | *p* |
| --- | --- | --- | --- | --- |
| + BF_Agree | 0.032 | | [-0.05, 0.11] | 0.45 |
| + BF_Con | 0.033 | | [-0.04, 0.11] | 0.44 |
| + BF_Es | 0.009 | | [-0.07, 0.09] | 0.83 |
| + BF_Ext | 0.032 | | [-0.04, 0.11] | 0.44 |
| + BF_Ope | 0.058 | | [-0.02, 0.13] | 0.15 |
| + PNQ | | 0.001 | [-0.07, 0.08] | 0.98 |
| + type of room [living] | 0.098 | | [-0.04, 0.24 | 0.18 |
| + Variation [non-climbing] | 0.071 | | [-0.10, 0.24] | 0.42 |
| **+ type of greenery [without greenery]** | **-0.129** | | **[-0.25, -0.004]** | **0.04** |
| **+ type of greenery [integrated greenery]** | **-0.179** | | **[-0.30, -0.05]** | **0.005** |
| + type of room *type of greenery [without greenery] | 0.098 | | [-0.07, 0.27] | 0.27 |
| **+ type of room *type of greenery [integrated greenery]** | **0.196** | | **[0.02, 0.37]** | **0.03** |
| +type of room [living]* Variation | -0.144 | | [-0.28, -0.00] | 0.05 |

**Table S7.** Post-hoc comparisons in Coherence.

| **Contrast** | **Estimate** | **SE** | **t-ratio** | **p-value** |
| --- | --- | --- | --- | --- |
| Bedroom with greenery - Living with greenery | -0.03 | 0.06 | -0.41 | 1.00 |
| Bedroom with greenery **-** Bedroom without greenery | 0.13 | 0.06 | 2.03 | 0.63 |
| Bedroom with greenery - Living without greenery | 0.00 | 0.06 | 0.07 | 1.00 |
| Bedroom with greenery - Bedroom integrated greenery | 0.18 | 0.06 | 2.83 | 0.07 |
| Bedroom with greenery - Living integrated greenery | -0.04 | 0.06 | -0.69 | 1.00 |
| Living with greenery **-** Bedroom without greenery | 0.16 | 0.06 | 2.45 | 0.22 |
| Living with greenery **-** Living without greenery | 0.03 | 0.06 | 0.48 | 1.00 |
| **Living with greenery - Bedroom integrated greenery** | **0.21** | **0.06** | **3.24** | **0.02** |
| Living with greenery - Living integrated greenery | -0.02 | 0.06 | -0.28 | 1.00 |
| Bedroom without greenery - Living without greenery | -0.12 | 0.06 | -1.97 | 0.74 |
| Bedroom without greenery **-** Bedroom integrated greenery | 0.05 | 0.06 | 0.79 | 1.00 |
| Bedroom without greenery **-** Living integrated greenery | -0.17 | 0.06 | -2.72 | 0.10 |
| Living without greenery **-** Bedroom integrated greenery | 0.17 | 0.06 | 2.76 | 0.09 |
| Living without greenery **-** Living integrated greenery | -0.05 | 0.06 | -0.76 | 1.00 |
| **Bedroom integrated greenery - Living integrated greenery** | **-0.22** | **0.06** | **-3.52** | **0.01** |

**Table S8.** Mixed-effect linear model for Compatibility

| *Compatibility* | β | | 95%CI | *p* |
| --- | --- | --- | --- | --- |
| + BF_Agree | -0.011 | | [-0.09, 0.07] | 0.78 |
| + BF_Con | -0.017 | | [-0.09, 0.06] | 0.68 |
| + BF_Es | 0.036 | | [-0.04, 0.11] | 0.41 |
| + BF_Ext | 0.015 | | [-0.06, 0.09] | 0.71 |
| + BF_Ope | -0.044 | | [-0.12, 0.03] | 0.28 |
| + PNQ | | -0.05 | [-0.13, 0.02] | 0.20 |
| **+ type of room [living]** | **0.150** | | **[0.009, 0.29]** | **0.04** |
| + Variation [non-climbing] | -0.076 | | [-0.24, 0.09] | 0.38 |
| **+ type of greenery [without greenery]** | **-0.304** | | **[-0.42, -0.18]** | **<.001** |
| + type of greenery [integrated greenery] | 0.102 | | [-0.01, 0.22] | 0.10 |
| + type of room *type of greenery [without greenery] | -0.045 | | [-0.21, 0.12] | 0.61 |
| + type of room *type of greenery [integrated greenery] | -0.172 | | [-0.34, 0.00] | 0.05 |
| +type of room [living]* Variation | -0.093 | | [-0.23, 0.04] | 0.20 |

**Table S9.** Mixed-effect linear model for Restful

| *Restful* | β | | 95%CI | *p* |
| --- | --- | --- | --- | --- |
| + BF_Agree | 0.034 | | [-0.04, 0.11] | 0.43 |
| + BF_Con | 0.001 | | [-0.08, 0.08] | 0.98 |
| + BF_Es | 0.024 | | [-0.06, 0.11] | 0.57 |
| + BF_Ext | 0.043 | | [-0.03, 0.12] | 0.30 |
| + BF_Ope | -0.020 | | [-0.10, 0.06] | 0.63 |
| + PNQ | | -0.022 | [-0.10, 0.05] | 0.59 |
| + type of room [living] | -0.046 | | [-0.18, 0.09] | 0.51 |
| **+ Variation [non-climbing]** | **-0.201** | | **[-0.37, -0.02]** | **0.02*** |
| **+ type of greenery [without greenery]** | **-0.295** | | **[-0.41, -0.17]** | **<.001** |
| + type of green [integrated greenery] | 0.028 | | [-0.09, 0.14] | 0.64 |
| + type of room *type of greenery [without greenery] | -0.086 | | [-0.25, 0.08] | 0.32 |
| + type of room *type of greenery [integrated greenery] | -0.057 | | [-0.22, 0.11] | 0.51 |
| +type of room [living]* Variation | 0.071 | | [-0.06, 0.20] | 0.32 |

**Table S10.** Mixed-effect linear model for Beautiful

| *Beautiful* | β | 95%CI | *p* |
| --- | --- | --- | --- |
| + BF_Agree | 0.008 | [-0.08, 0.09] | 0.84 |
| + BF_Con | 0.007 | [-0.08, 0.09] | 0.87 |
| + BF_Es | 0.023 | [-0.06, 0.11] | 0.59 |
| + BF_Ext | 0.080 | [-0.00, 0.16] | 0.06 |
| + BF_Ope | -0.026 | [-0.11, 0.05] | 0.52 |
| + PNQ | -0.009 | [-0.09, 0.07] | 0.82 |
| + type of room [living] | 0.091 | [-0.04, 0.23] | 0.19 |
| **+ Variation [non-climbing]** | **-0.208** | **[-0.38, -0.04]** | **0.02** |
| **+ type of greenery [without greenery]** | **-0.353** | **[-0.47, -0.24]** | **<.001** |
| + type of greenery [integrated greenery] | 0.059 | [-0.06, 0.18] | 0.33 |
| + type of room *type of green [without green] | 0.029 | [-0.14, 0.20] | 0.73 |
| + type of room *type of greenery [integrated greenery] | -0.025 | [-0.19, 0.14] | 0.77 |
| +type of room [living]* Variation | -0.043 | [-0.18, 0.09] | 0.53 |

**Table S11.** Mixed-effect linear model for Inviting

| *Inviting* | β | 95%CI | *p* |
| --- | --- | --- | --- |
| + BF_Agree | 0.035 | [-0.04, 0.11] | 0.39 |
| + BF_Con | 0.001 | [-0.07, 0.08] | 0.98 |
| + BF_Es | 0.038 | [-0.04, 0.12] | 0.37 |
| + BF_Ext | 0.029 | [-0.05, 0.10] | 0.48 |
| + BF_Ope | -0.049 | [-0.12, 0.02] | 0.22 |
| + PNQ | -0.033 | [-0.11, 0.04] | 0.42 |
| **+ type of room [living]** | **0.024** | **[0.10, 0.38]** | **<.001** |
| + Variation [non-climbing] | -0.097 | [-0.16, 0.16] | 0.99 |
| **+ type of greenery [without greenery]** | **-0.038** | **[-0.50, -0.26]** | **<.001** |
| + type of green [integrated greenery] | 0.094 | [-0.02, 0.21] | 0.13 |
| + type of room *type of greenery [without greenery] | -0.055 | [-0.22, 0.11] | 0.53 |
| + type of room *type of greenery [integrated greenery] | -0.017 | [-0.34, -0.00] | 0.05 |
| **+type of room [living]*** **Variation** | **-0.015** | **[-0.29, -0.01]** | **0.03** |

**Table S12.** Post-hoc comparisons between Bedroom and Living rooms for Inviting.

| **Contrast** | **Conparison** | **Differences** | **SE** | **t-ratio** | **p-value** |
| --- | --- | --- | --- | --- | --- |
| **Climbing greenery** | **Bedroom - Living** | **-0.16543** | **0.051** | **-3.246** | **0.0012** |
| **Non-climbing greenery** | Bedroom - Living | -0.00713 | 0.051 | -0.140 | 0.8888 |

**Table S13.** Mixed-effect linear model for Lively

| *Lively* | β | 95%CI | *p* |
| --- | --- | --- | --- |
| + BF_Agree | 0.057 | [-0.02, 0.13] | 0.17 |
| + BF_Con | 0.041 | [-0.03, 0.12] | 0.32 |
| + BF_Es | 0.037 | [-0.04, 0.12] | 0.37 |
| + BF_Ext | 0.062 | [-0.01, 0.14] | 0.12 |
| + **BF_Ope** | **-0.118** | **[-0.19, -0.04]** | **0.003** |
| + PNQ | -0.036 | [-0.11, 0.04] | 0.37 |
| **+ type of room [living]** | **0.181** | **[0.04, 0.31]** | **0.009** |
| + Variation [non-climbing] | -0.047 | [-0.21, 0.11] | 0.57 |
| **+ type of greenery [without greenery]** | **-0.404** | **[-0.52, -0.28]** | **<.001** |
| + type of greenery [integrated greenery] | 0.109 | [-0.008, 0.22] | 0.07 |
| + type of room *type of greenery [without greenery] | -0.057 | [-0.22, 0.10] | 0.49 |
| + type of room *type of greenery [integrated greenery] | -0.047 | [-0.21, 0.11] | 0.58 |
| +type of room [living]* Variation | -0.119 | [-0.25, 0.01] | 0.08 |

**Table S14.** Mixed-effect linear model for Chaotic

| *Chaotic* | β | 95%CI | *p* |
| --- | --- | --- | --- |
| + BF_Agree | -0.034 | [-0.10, 0.03] | 0.36 |
| + BF_Con | -0.042 | [-0.11,- 0.03] | 0.26 |
| + BF_Es | -0.031 | [-0.10, 0.04] | 0.41 |
| + BF_Ext | -0.036 | [-0.10, 0.03] | 0.32 |
| **+ BF_Ope** | **-0.139** | **[-0.20, -0.06]** | **0.0001** |
| **+ PNQ** | **-0.084** | **[-0.15, -0.01]** | **0.02** |
| + type of room [living] | 0.141 | [-0.01, 0.29] | 0.07 |
| + Variation [non-climbing] | 0.010 | [-0.14, 0.16] | 0.90 |
| + type of greenery [without greenery] | 0.012 | [-0.12, 0.14] | 0.85 |
| **+ type of greenery [integrated greenery]** | **0.144** | **[0.01, 0.27]** | **0.03** |
| + type of room *type of greenery [without greenery] | -0.131 | [-0.31, 0.05] | 0.17 |
| + type of room *type of greenery [integrated greenery] | -0.178 | [-0.36, 0.008] | 0.06 |
| **+type of room [living]*** **Variation** | **-0.228** | **[-0.38, -0.07]** | **0.004** |

**Table S15.** Post-hoc comparisons between Bedroom and Living rooms for Chaotic

| **Contrast** | **Conparison** | **Differences** | **SE** | **t-ratio** | **p-value** |
| --- | --- | --- | --- | --- | --- |
| **Climbing greenery** | Bedroom - Living | -0.03 | 0.05 | -0.68 | 0.49 |
| **Non-climbing greenery** | **Bedroom - Living** | **0.19** | **0.05** | **3.43** | **0.0006** |

**Table S16.** Mixed-effect linear model for Oppressive

| *Oppressive* | β | 95%CI | *p* |
| --- | --- | --- | --- |
| + BF_Agree | -0.070 | [-0.15, 0.01] | 0.09 |
| + BF_Con | -0.045 | [-0.12, 0.03] | 0.28 |
| + BF_Es | -0.032 | [-0.11, 0.05] | 0.45 |
| + BF_Ext | -0.022 | [-0.10, 0.05] | 0.58 |
| + BF_Ope | -0.014 | [-0.09, 0.06] | 0.71 |
| + PNQ | -0.019 | [-0.09, 0.05] | 0.64 |
| + type of room [living] | 0.038 | [-0.10, 0.18] | 0.59 |
| **+ Variation [non-climbing]** | **0.239** | **[0.07, 0.40]** | **0.006*** |
| **+ type of greenery [without greenery]** | **0.378** | **[0.25, 0.50]** | **<.001** |
| + type of greenery [integrated greenery] | 0.022 | [-0.10, 0.14] | 0.72 |
| + type of room *type of greenery [without greenery] | -0.126 | [-0.30, 0.04] | 0.16 |
| + type of room *type of greenery [integrated greenery] | -0.022 | [-0.19, 0.15] | 0.80 |
| +type of room [living]*Variation | -0.101 | [-0.24, 0.04] | 0.16 |

**Table S17.** Mixed-effect linear model for Monotonous

| *Monotonous* | β | 95%CI | *p* |
| --- | --- | --- | --- |
| + BF_Agree | -0.034 | [-0.11, 0.04] | 0.38 |
| + BF_Con | -0.050 | [-0.12, 0.02] | 0.20 |
| + BF_Es | -0.021 | [-0.09, 0.05] | 0.59 |
| + BF_Ext | -0.007 | [-0.08, 0.06] | 0.85 |
| + BF_Ope | 0.062 | [-0.01, 0.13] | 0.10 |
| + PNQ | 0.027 | [-0.04, 0.10] | 0.47 |
| **+ type of room [living]** | **-0.214** | **[-0.36, -0.06]** | **0.004** |
| + Variation [non-climbing] | 0.011 | [-0.15, 0.17] | 0.89 |
| **+ type of greenery [without greenery]** | **0.368** | **[0.24, 0.49]** | **<.001** |
| **+ type of greenery [integrated greenery]** | **-0.137** | **[-0.26, -0.009]** | **0.04** |
| + type of room *type of greenery [without greenery] | 0.054 | [-0.12, 0.23] | 0.55 |
| + type of room *type of greenery [integrated greenery] | 0.144 | [-0.03, 0.32] | 0.12 |
| **+type of room [living]*Variation** | **0.191** | **[0.04, 0.33]** | **0.01** |

**Table S18.** Post-hoc comparisons between Bedroom and Living rooms for Monotonous.

| **Contrast** | **Conparison** | **Differences** | **SE** | **t-ratio** | **p-value** |
| --- | --- | --- | --- | --- | --- |
| **Climbing greenery** | **Bedroom - Living** | **0.14** | **0.05** | **2.78** | **0.0054** |
| **Non-climbing greenery** | Bedroom - Living | -0.04 | 0.05 | -0.82 | 0.4092 |

**Figures S1- S11**

Means’ distributions of restorativeness and affective qualities of rooms


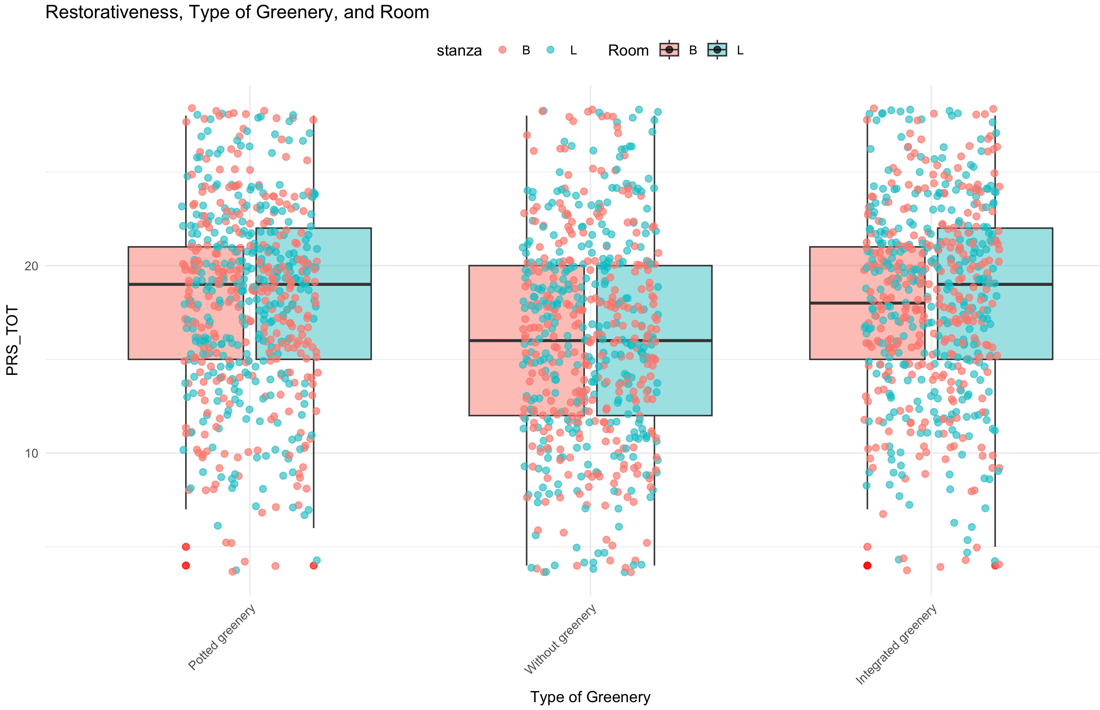


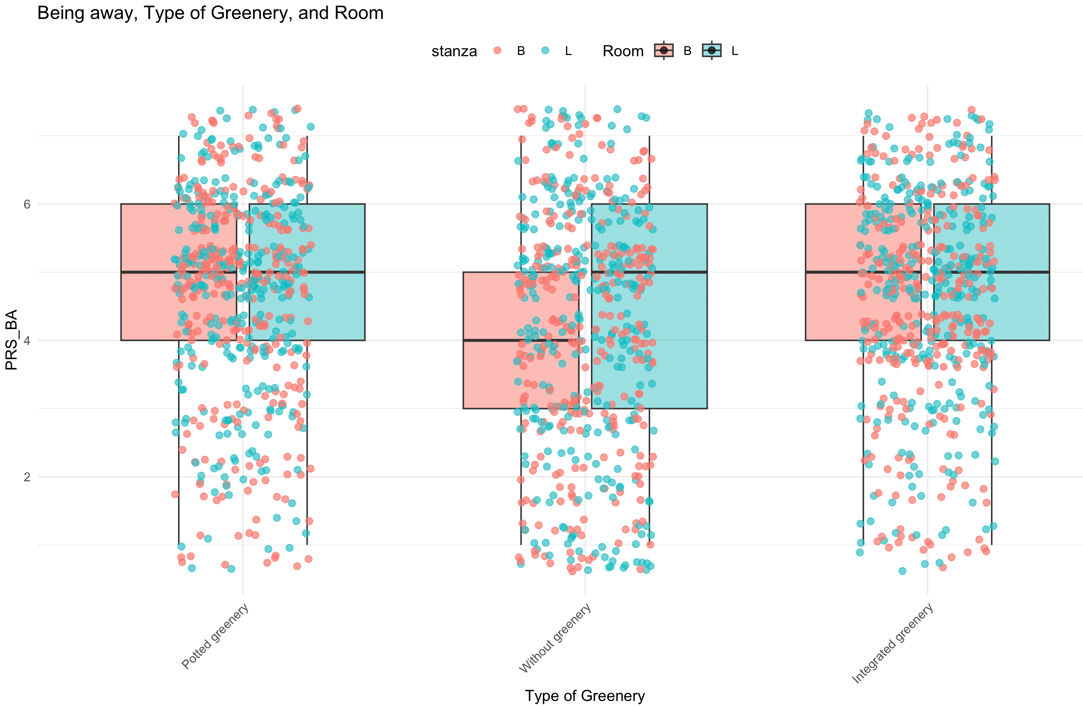


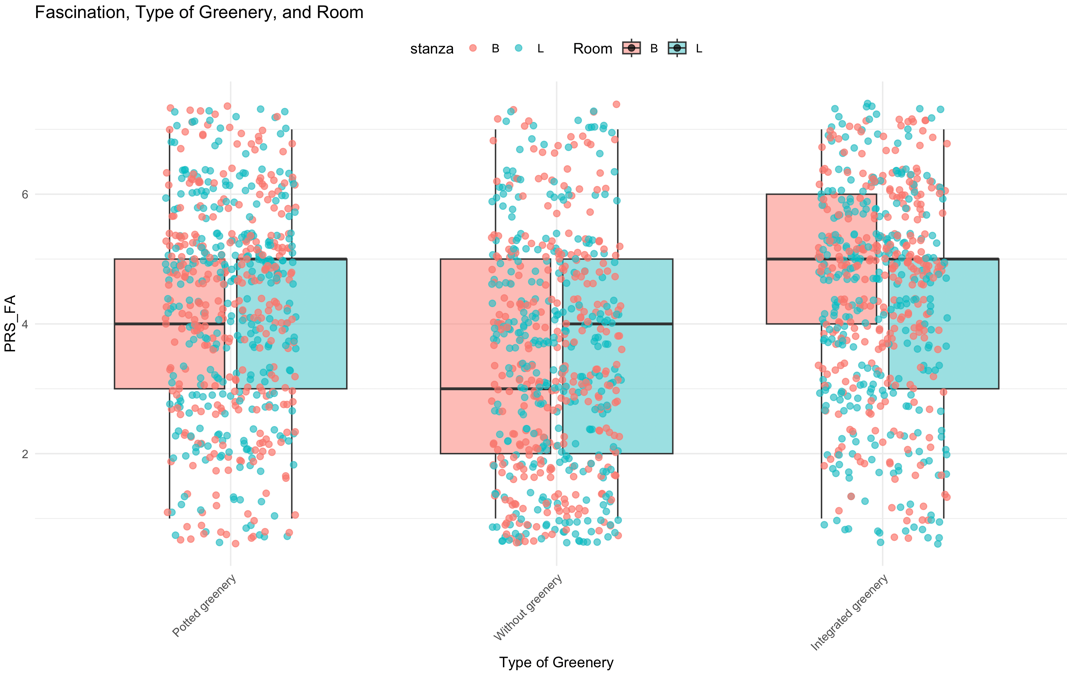


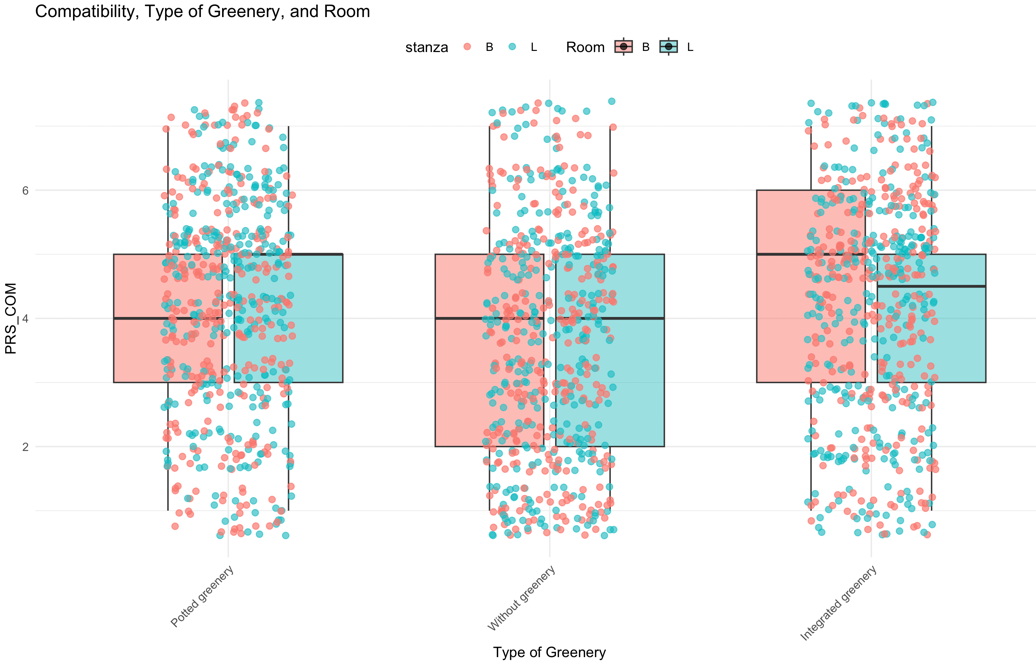


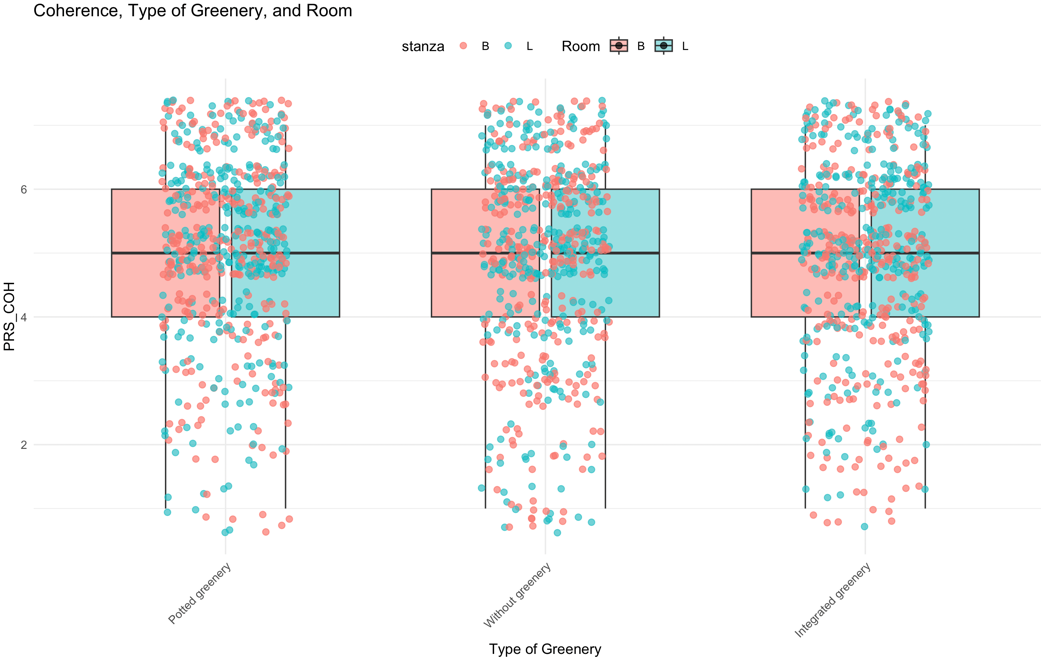


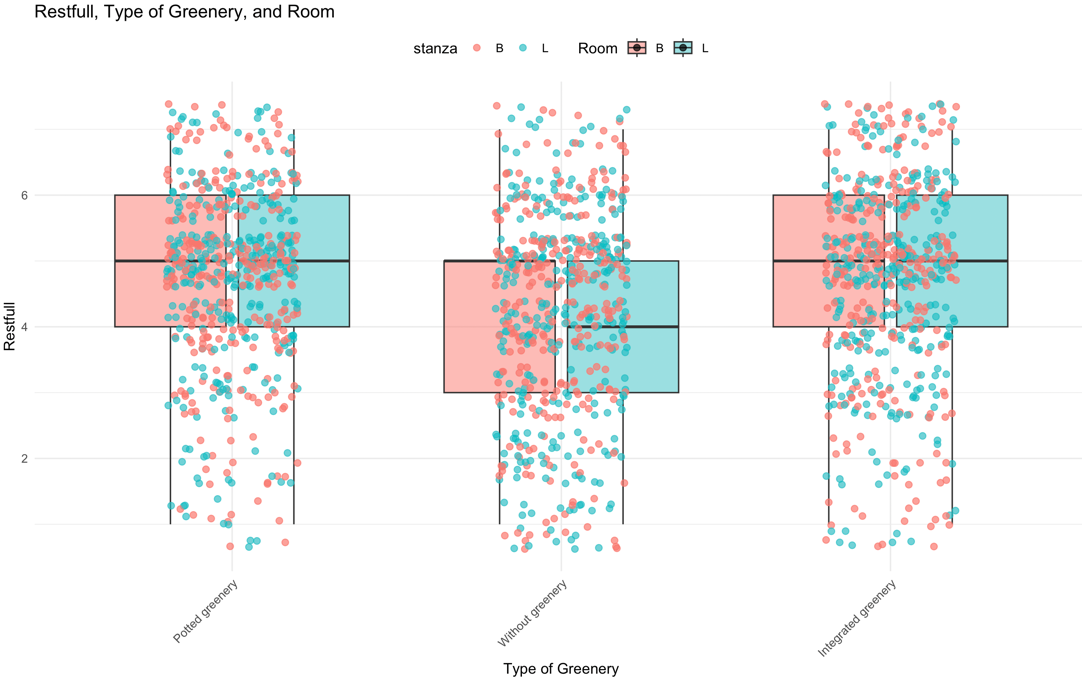


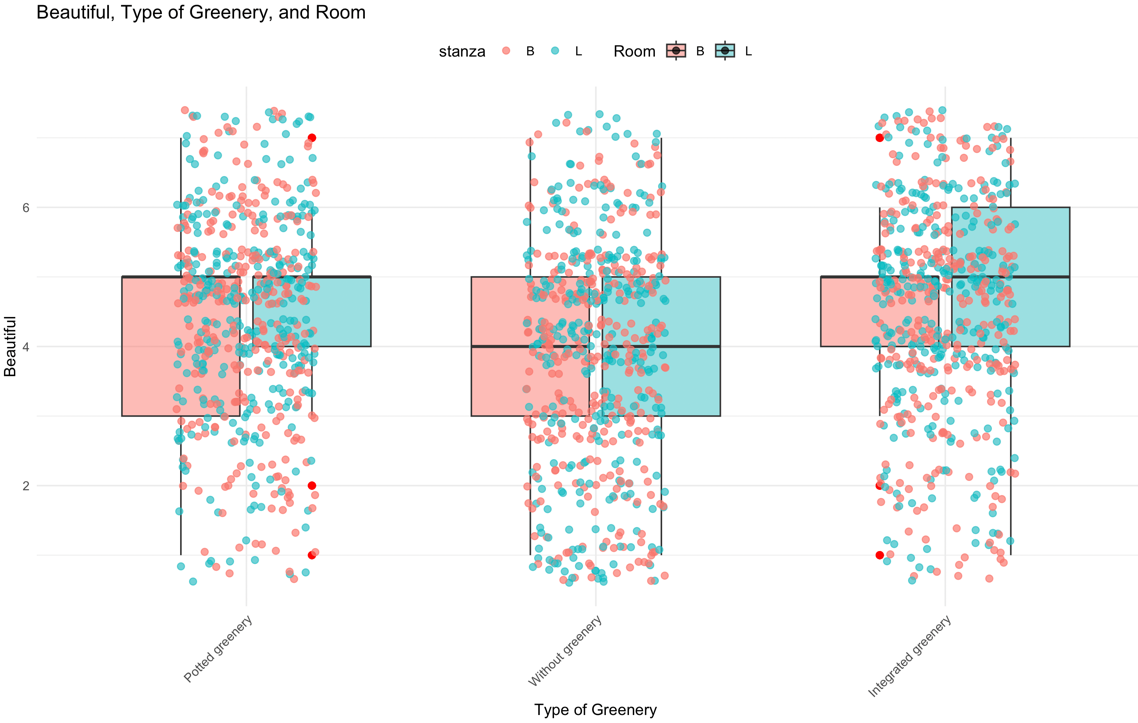


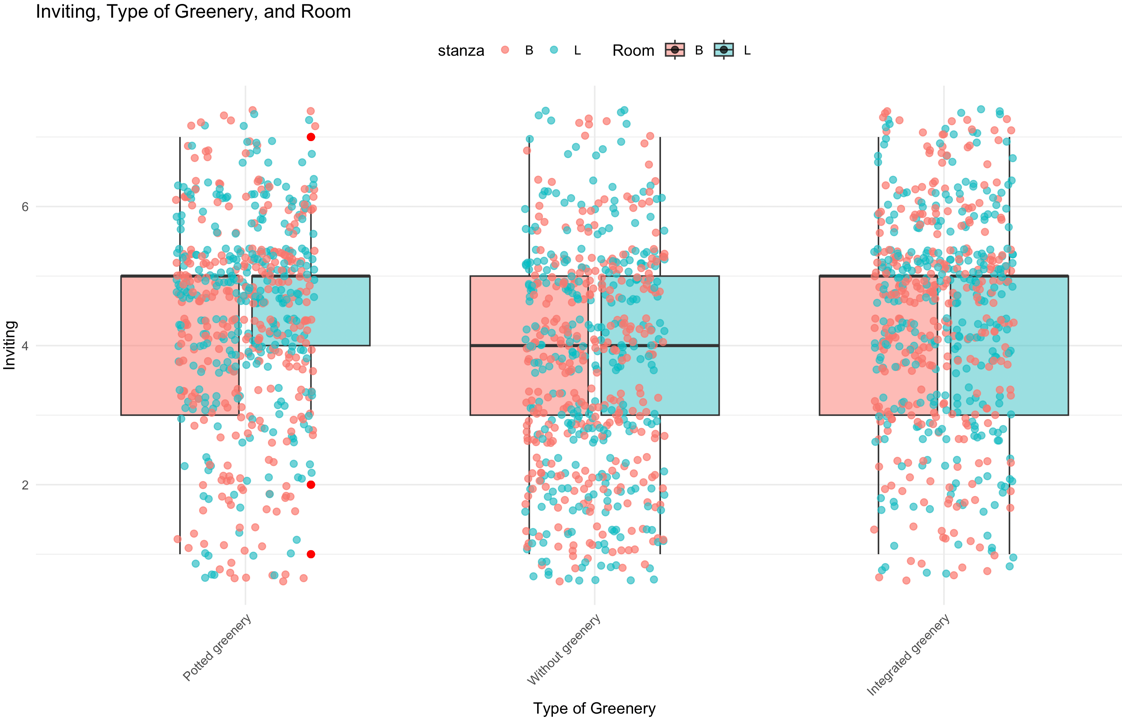


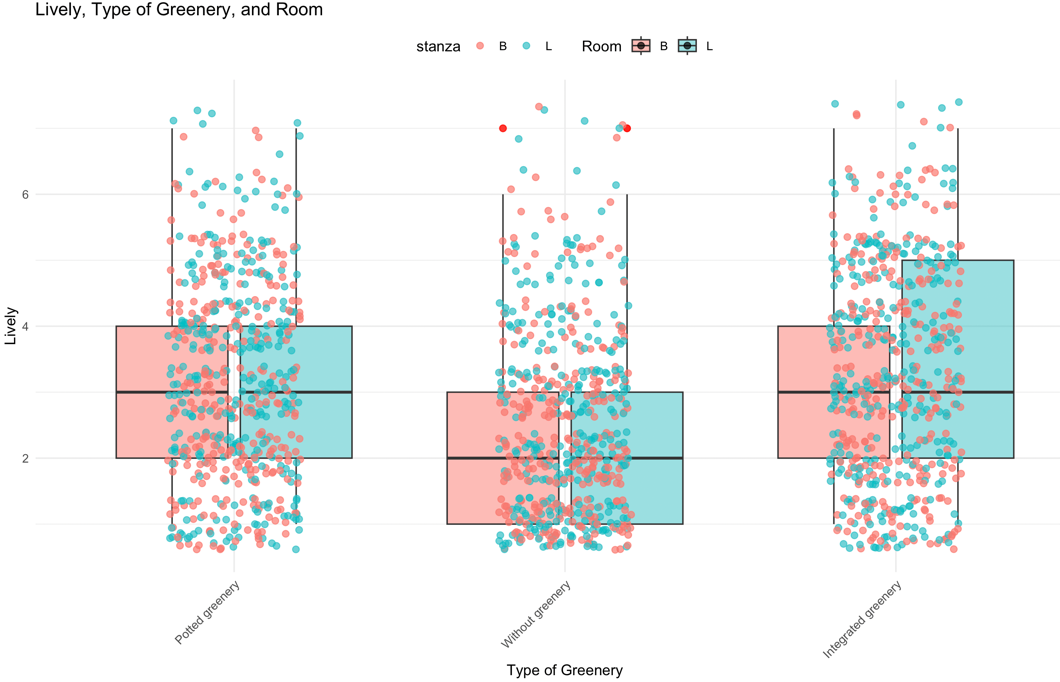


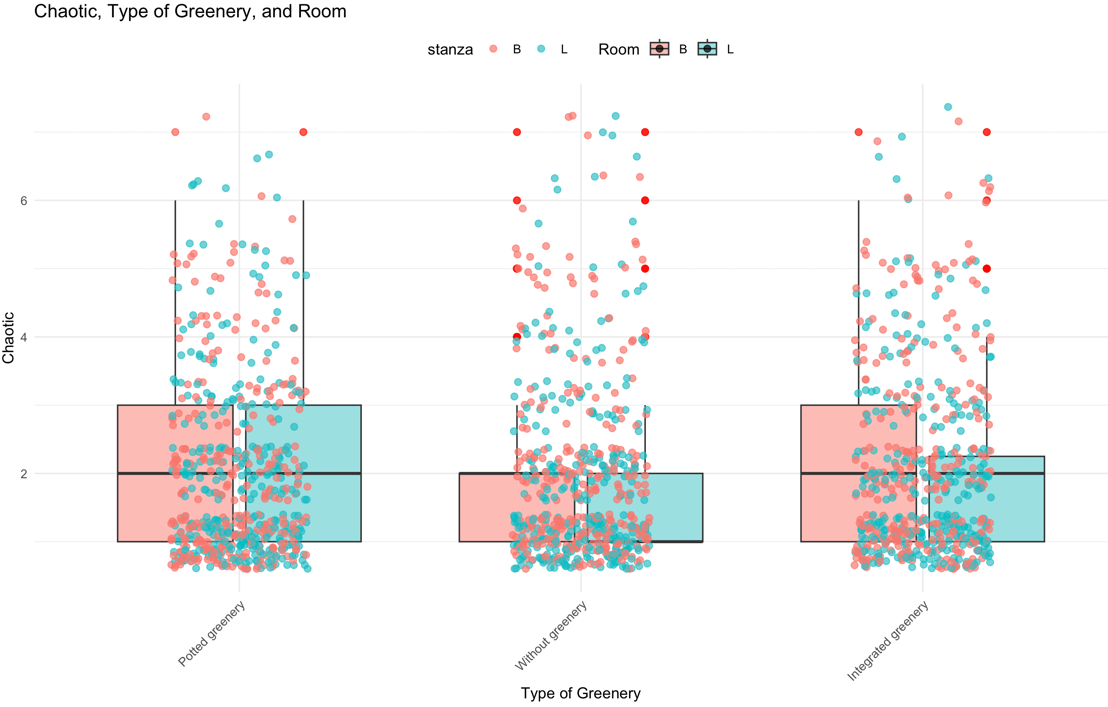


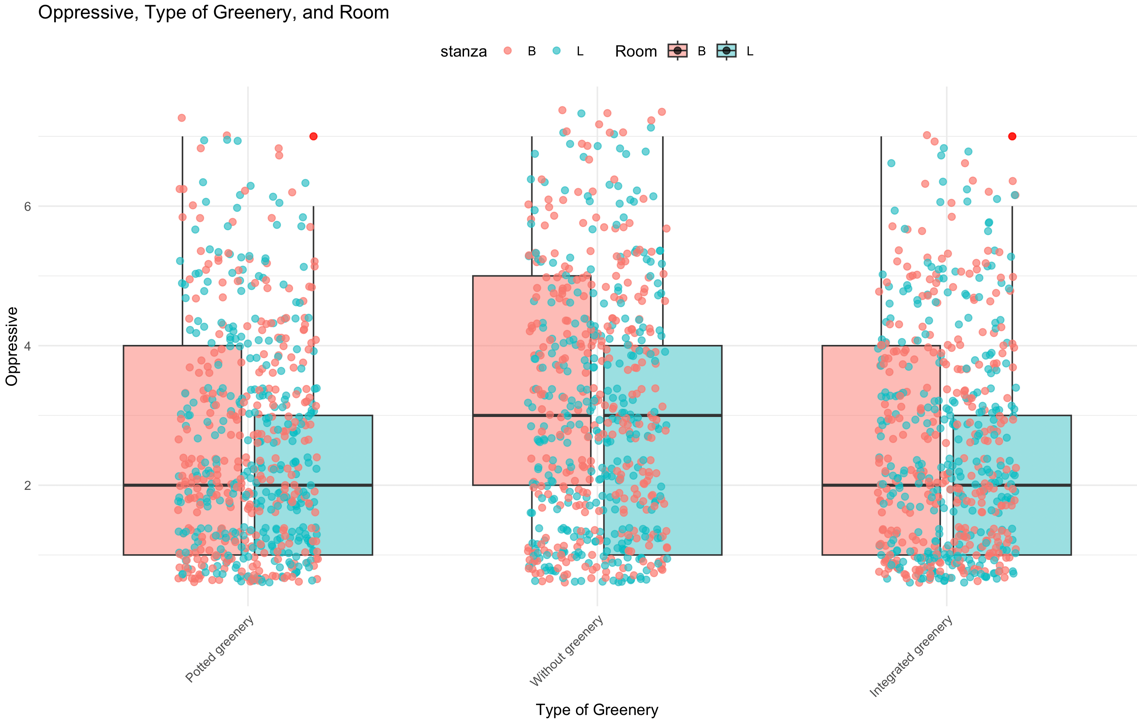


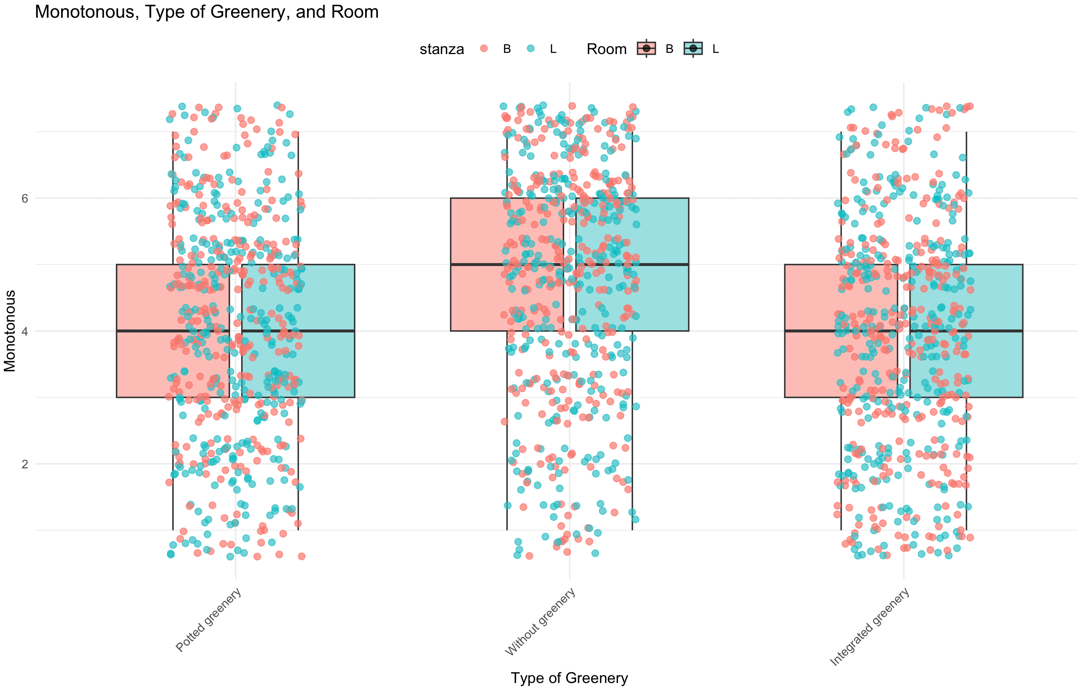

Supplement: Supplementary file 1 [file Table_1.docx]
